# Machine Learning for the ECG Diagnosis and Risk Stratification of Occlusion Myocardial Infarction at First Medical Contact

**DOI:** 10.21203/rs.3.rs-2510930/v1

**Published:** 2023-01-30

**Authors:** Salah Al-Zaiti, Christian Martin-Gill, Jessica Zégre-Hemsey, Zeineb Bouzid, Ziad Faramand, Mohammad Alrawashdeh, Richard Gregg, Stephanie Helman, Nathan Riek, Karina Kraevsky-Phillips, Gilles Clermont, Murat Akcakaya, Susan Sereika, Peter Van Dam, Stephen Smith, Yochai Birnbaum, Samir Saba, Ervin Sejdic, Clifton Callaway

**Affiliations:** University of Pittsburgh; University of Pittsburgh; University of North Carolina; University of Pittsburgh; Northeast Georgia Health System; Harvard Medical School; Philips (United States); University of Pittsburgh; University of Pittsburgh; University of Pittsburgh; University of Pittsburgh; University of Pittsburgh; University of Pittsburgh; University Medical Center Utrecht; Hennepin Healthcare and University of Minnesota; Baylor College of Medicine; University of Pittsburgh; University of Toronto; University of Pittsburgh

## Abstract

Patients with occlusion myocardial infarction (OMI) and no ST-elevation on presenting ECG are increasing in numbers. These patients have a poor prognosis and would benefit from immediate reperfusion therapy, but we currently have no accurate tools to identify them during initial triage. Herein, we report the first observational cohort study to develop machine learning models for the ECG diagnosis of OMI. Using 7,313 consecutive patients from multiple clinical sites, we derived and externally validated an intelligent model that outperformed practicing clinicians and other widely used commercial interpretation systems, significantly boosting both precision and sensitivity. Our derived OMI risk score provided superior rule-in and rule-out accuracy compared to routine care, and when combined with the clinical judgment of trained emergency personnel, this score helped correctly reclassify one in three patients with chest pain. ECG features driving our models were validated by clinical experts, providing plausible mechanistic links to myocardial injury.

## Introduction

The ECG diagnosis of acute coronary syndrome (ACS) in patients with acute chest pain is a longstanding challenge in clinical practice.^1–5^ Guidelines primarily focus on ST-segment elevation (STE) for discerning patients with ST-elevation myocardial infarction (STEMI) vs. other forms of ACS.^6–9^ A biomarker-driven approach is recommended in the absence of STE on the presenting ECG. This diagnostic paradigm has two important limitations. First, around 24%−35% of patients with non-STEMI have total coronary occlusion, referred to as occlusion myocardial infarction (OMI), and require emergent catheterization.^10–14^ This vulnerable group, in contrast to acute myocardial infarction with an open artery (non-OMI) (**Extended Data Fig. 1**), suffers from unnecessary diagnostic and treatment delays that are associated with higher mortality.^15–18^ This excess risk can be mitigated with enhanced diagnostic criteria. Although important ECG signatures of OMI are frequently described in the literature,^19–22^ they are subtle, involve the entire QRST complex, and are spatial in nature (i.e., changes diluted across multiple leads).^23–26^ Visual inspection of ECG images by clinical experts, thus, is suboptimal and leads to a high degree of variability in ECG interpretation.^27–29^

The second limitation is that cardiac biomarkers, including conventional or high sensitivity troponin (hs-cTn), cannot differentiate OMI until peak level is reached, which is too late to salvage myocardium. Positive troponin results (>99^th^ percentile limit) come with a high false positive rate, and approximately one-third of patients remain in a biomarker-indeterminate “observation zone” even after serial sampling.^30,31^ More importantly, ~25% of acute myocardial infarction cases have a negative initial hs-cTn, which is observed in both the STEMI and OMI subgroups.^32^ Consequently, 25%−30% of patients with OMI are not treated in a timely fashion, and around 63% (IQR 38%−81 %) of patients evaluated for chest pain at the emergency department are admitted to the hospital because of an inconclusive initial assessment.^33^ These diagnostic limitations have created a costly, inefficient clinical practice paradigm where most patients with chest pain are over-monitored while some patients with OMI have delayed diagnosis and treatment, potentially contributing to the 14%−22% excess risk of mortality seen in the non-STE ACS group (NSTE-ACS).^16,34,35^

Herein, we describe the first multisite, prospective, observational cohort study to evaluate the diagnostic accuracy of machine learning for the ECG diagnosis and risk stratification of OMI at first medical contact in an observer-independent approach **(Extended Data Fig. 2)**. Our intelligent models were derived and externally validated on 7,313 patients with chest pain from multiple clinical sites in the United States. The results demonstrate the superiority of machine learning in detecting subtle ischemic ECG changes indicative of OMI, outperforming practicing clinicians and other widely used commercial ECG interpretation software. Our derived OMI risk score provides superior rule-in and rule-out accuracy when compared to the HEART score, helping correctly reclassify one in three patients with chest pain. We identified the most important ECG features driving our model’s classifications and identified plausible mechanistic links to myocardial injury.

## Results

### Sample Characteristics

**Figure 1:**
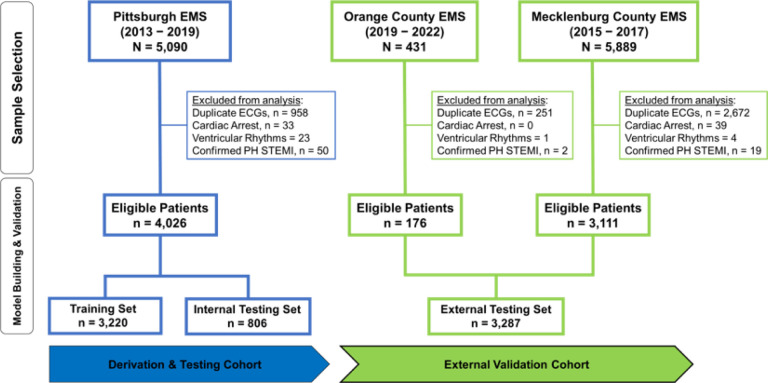
Cohort and sample selection This flow diagram shows patient inclusion and exclusion in each cohort, as well as the dataset partition for training, internal testing, and external validation. Exclusions are not mutually exclusive.

After excluding patients with cardiac arrest, ventricular tachyarrhythmias, confirmed prehospital STEMI, and duplicate ECGs, our derivation cohort included 4,026 consecutive patients with chest pain (age 59 ± 16 years, 47% females, 5.2% OMI). The two external validation cohorts together included 3,287 patients (age 60 ± 15 years, 45% females, 6.4% OMI) (Fig. 1 and Table 1). Most patients in the derivation and validation cohorts were in normal sinus rhythm (> 80%) and around 10% were in atrial fibrillation. Around 3% of patients had left bundle branch block (LBBB) and ~ 10% had ECG-evidence of left ventricular hypertrophy (LVH). The derivation and validation cohorts were comparable in terms of age, sex, baseline clinical characteristics, and 30-day cardiovascular mortality. The validation cohort, however, had more Black and Hispanic minorities and a slightly higher rate of ACS and OMI. The presence of OMI, defined as a culprit coronary artery with a TIMI flow grade of 0–1, was adjudicated from charts by independent reviewers blinded to all ECG analyses. A TIMI flow grade of 2 with significant coronary narrowing (>70%) and peak 4th generation (not high sensitivity) troponin of 5–10 ng/mL was also indicative of OMI.

### Algorithm Derivation and Testing

Input data for model training was based on prehospital 12-lead ECGs obtained at first medical contact. We selected 73 morphological ECG features out of 554 temporal-spatial metrics using a hybrid data-driven and domain expertise approach.^19^ Using these features, ten classifiers were trained to learn ischemic patterns between ACS and non-ACS groups and to estimate the probability of OMI: regularized logistic regression, linear discriminant analysis, support vector machine, Gaussian NaTve Bayes, random forest, gradient boosting machine, extreme gradient boosting, stochastic gradient descent logistic regression, k-nearest neighbors, and artificial neural networks. We chose these classifiers because they learn different mathematical representations in the data, maximizing the chance of finding the best modeling approach for relating complex ECG data to underlying physiology.

**Figure 2:**
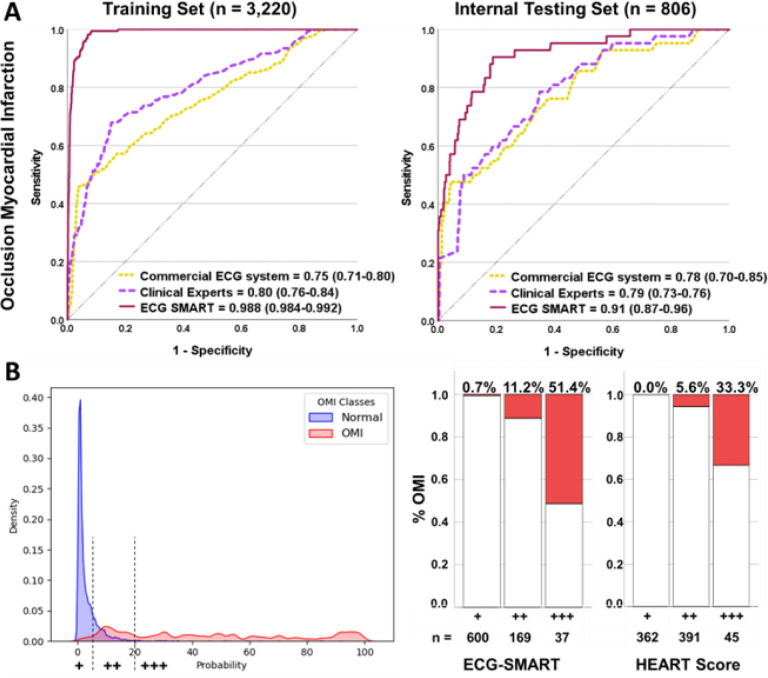
Algorithm derivation and testing This figure shows (A) the classification performance of the machine learning model against other reference standards for detecting occlusion myocardial infarction (OMI), (B) the probability density plots of OMI(+) and OMI(−) classes as denoted by the machine learning model, along with optimal cutoffs of low-risk, intermediate, and high-risk, and (C) distribution of patients in low-risk (+), intermediate risk (++) and high-risk (+++) as per the machine learning model and HEART score.

The random forest model achieved the best bias-variance tradeoff for training and internal testing. We compared the random forest against the ECG interpretation of practicing clinicians and against the performance of a commercial ECG interpretation system that is FDA-cleared for “Acute MI” diagnosis. On the hold-out test set, the random forest model (AUROC 0.91 [95% CI 0.87–0.96]) outperformed both practicing clinicians (AUROC 0.79 [95% CI 0.73–0.76], p < 0.001) and the commercial ECG system (AUROC 0.78 [95% CI 0.70–0.85], p< 0.001) (Fig. 2A).

Next, we used probability density plots for OMI(+) and OMI(−) classes to denote the optimal separation margins for risk prediction. As recommended by guidelines,^7^ we defined a risk score to identify patients at low risk (OMI score< 5), intermediate risk (OMI score 5–20), and high risk (OMI score >20), with these cutoffs yielding excellent separation between classes (Log-rank chi-square 133.04, df = 2, p < 0.001) (Fig. 2B, **left panel**). Our OMI score classified 74.4% of patients as low-risk and 4.6% as high-risk. Using the low-risk group in a rule-out strategy yielded a sensitivity of 0.91 and a negative predictive value (NPV) of 0.993, with an overall missed event rate of 0.5%. Using high-risk class for a rule-in strategy yielded a specificity of 0.976 and a positive predictive value (PPV) of 0.514, with an overall false discovery rate of 2%. Finally, we compared this OMI score to the HEART score, which uses patient history, ECG data, age, risk factors, and troponin values (Fig. 2B, **right panel**). Our OMI score, which is based on ECG data alone, classified 66% more patients as low risk than the HEART score with a comparable false negative rate < 1 %, and classified fewer patients as high-risk and with much higher precision (51 % vs. 33%). The OMI score also triaged 50% fewer patients as intermediate risk and still got better discrimination for OMI detection (11.2% vs. 5.6%).

### Model Explainability

**Figure 3:**
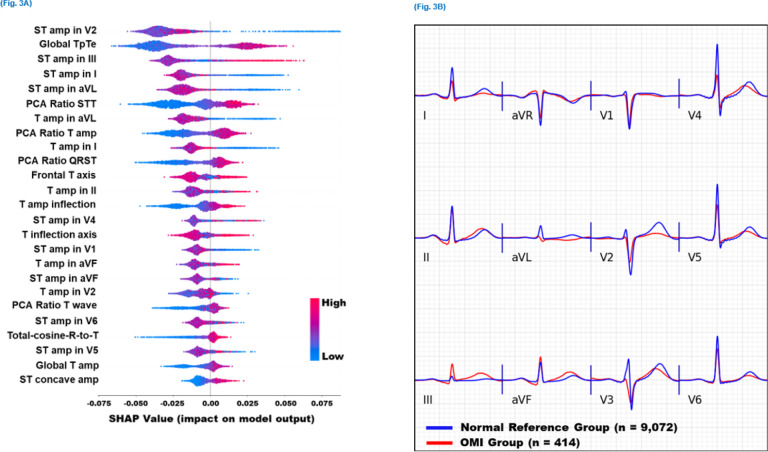
Model explainability for OMI detection This figure shows (A) SHAP values for the 25 most important features driving the predictions of the machine learning classifier in the derivation cohort, and (B) the aggregate median beats of ECGs with occlusion myocardial infarction (OMI) class (red) and the aggregate median beats of ECGs with normal sinus rhythm and no OMI (blue).

We used Tree SHAP algorithms to generate an importance ranking that explains the output of the random forest model based on SHAP values estimated for the top 25 features (Fig. 3A). The features with the greatest impact on classification output included slight ST-depression in leads V1, V2, I, and aVL; slight ST-elevation in leads III and V4-V6; loss of concave pattern in anterior leads; T wave enlargement in II and aVF and T flattening or inversion in I and aVL; prolonged T_peak_-T_end_ interval; T axis deviation; increased repolarization dispersion; and distorted directions of activation and recovery patterns. Most of these ECG patterns can be mechanistically linked to cardiac ischemia, suggesting their clinical value as plausible features for OMI detection.

To better visualize these global ECG patterns detected by our model, we created pooled population median beats for the OMI(+) class (n = 414 ECGs), and superimposed these median beats on the pooled population median beats of patients with normal sinus rhythm and OMI(−) status (n = 9,072 ECGs) (Fig. 3B). Findings from this figure agree with the patterns derived from the SHAP values described above. Specifically, this figure illustrates that OMI is associated with ST-depression and T flattening in V1-V2, I, and aVL; slight ST-elevation in the anterior leads with loss in concave pattern; peaked T wave in inferior leads; T_peak_-T_end_ prolongation (seen in many leads); global repolarization dispersion (seen as peaked T in some leads and flattening in others); T axis deviation (away from the left ventricle), and distorted activation and recovery patterns (seen in the horizontal plane as loss of R wave progression in precordial leads with increased T wave discordance). Due to prevalent multivessel disease in this cohort, these OMI patterns remained relatively consistent regardless of culprit location.

### External Validation

**Figure 4:**
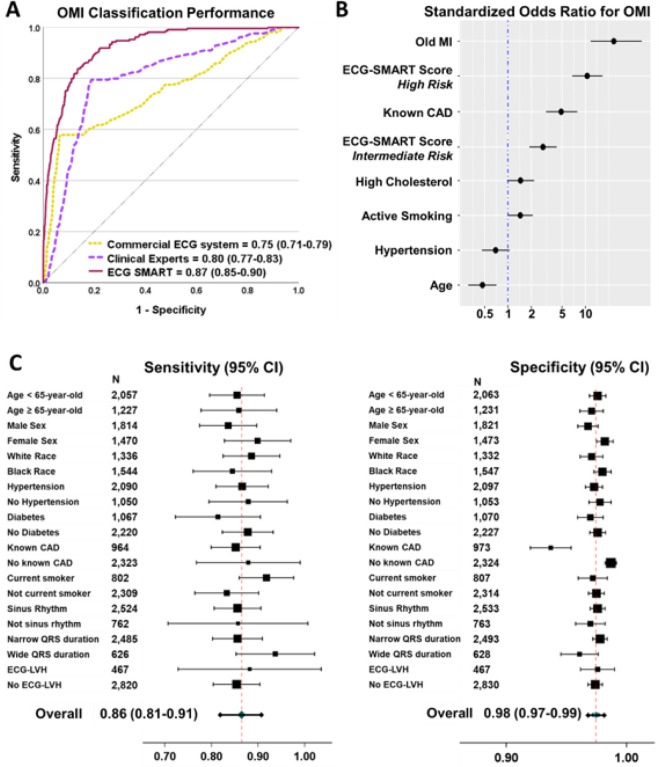
External validation of ECG-SMART algorithm This figure shows (A) the classification performance of the machine learning model against other reference standards for detecting occlusion myocardial infarction (OMI), (B) the independent clinical predictors of OMI on multivariate logistic regression testing, and (C) the overall sensitivity and specificity (95% confidence interval [CI]) of the derived OMI score, along with breakdown across subgroups based on age, sex, comorbidities, and baseline ECG findings. The size of markers denotes the sample size of the respective subgroup.

We tested the final lock-out model on 3,287 patients from two independent external clinical sites. Machine learning engineers were blinded to outcome data from other sites, and the pre-populated model predictions were independently evaluated by the clinical investigators. Our model generalized well and maintained high classification performance (AUROC 0.873 [95% CI 0.85–0.90]), outperforming the classification performance of the commercial ECG system (AUROC 0.75 [95% CI 0.71 −0.79], p < 0.001) and practicing clinicians (AUROC 0.80 [95% CI 0.77–0.83], p < 0.001) (Fig. 4A). Our OMI risk score was a strong predictor of OMI, independent from, age, sex, and other coronary risk factors (OR 10.6 [95% CI 6.78–16.64] for high-risk class and OR 2.85 [95% CI 1.91 −4.28] for intermediate-risk class) (Fig. 4B). This risk score triaged 69% of patients in the low-risk group at a false-negative rate of 1.3% and identified 5.1 % of patients as high-risk at acceptable true positive rate > 50%. The overall sensitivity, specificity, PPV, and NPV for the OMI rule-in and rule-out strategy were 0.86 (95% CI 0.81–0.91), 0.98 (95% CI 0.97–0.99), 0.54 (95% CI 0.46–0.62), and 0.99 (95% CI 0.98–0.99), respectively. This diagnostic accuracy remained relatively similar across subgroups based on age, sex, comorbidities, and baseline ECG findings, indicating the lack of aggregation bias (Fig. 4C). In comparison, the sensitivity, specificity, PPV, and NPV for ECG overread by practicing clinicians were 0.58, 0.93, 0.36, and 0.97, and for the commercial ECG system 0.79, 0.80, 0.22, and 0.98, respectively.

**Figure 5:**
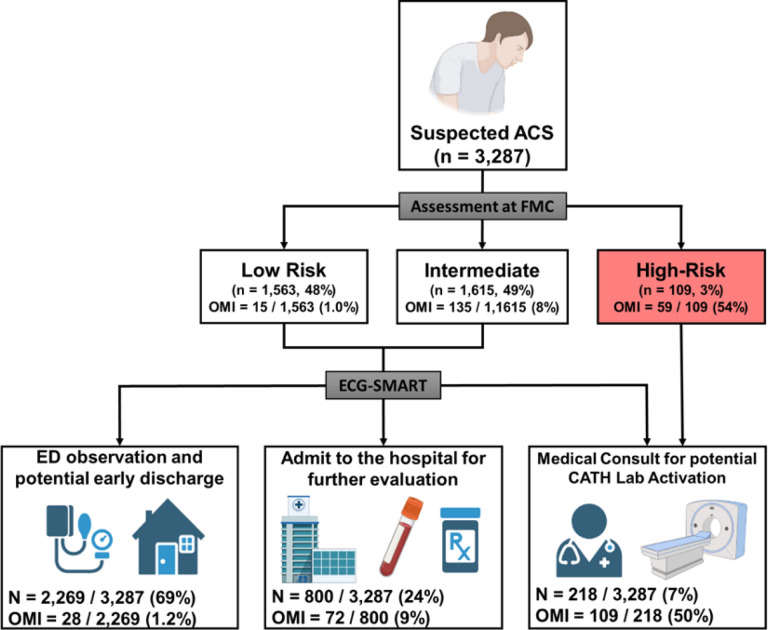
Decision analysis for the incremental gain of OMI risk score in reclassifying patients This figure simulates the incremental gain of the derived risk score in reclassifying the initial triage decisions by emergency personnel at first medical contact.

Next, we used decision analysis to evaluate the incremental gain of our derived risk score in re-classifying patients at first medical contact (Fig. 5). To simulate initial assessment by emergency personnel, we used the modified HEAR score (History, ECG, Age, and Risk factors) to triage patients into low, intermediate, and high-risk groups. At baseline, emergency personnel triaged 48% of patients as low risk with a NPV of 99.0% and triaged 3% of patients as high risk with a PPV of 54.1 %. Nearly 50% of patients remained in an indeterminate observation zone. Applying our OMI risk score would help triage 45% more patients as low risk while keeping the NPV at 98.8% and would help detect 85% more cases with OMI while keeping PPV at 50.0%. The OMI score would also help reduce the number of patients in the indeterminate observation zone by more than half. These numbers translate into a net reclassification improvement (NRI) index of 41 % (95% CI 33%−50%). To validate this incremental clinical utility, we manually reviewed ECGs reclassified correctly as OMI(+) (**Extended data** Fig. 3). Many of these ECGs showed subtle or nonspecific changes that were nondiagnostic as per guidelines,^6^ suggesting potential value in boosting provider’s confidence when interpreting “fuzzy” ECGs.

Finally, we investigated the potential sources of false negatives in the validation data. Among those with missed OMI events (n = 28, 0.9%), many patients had high-frequency noise and baseline wander on their initial ECG (n = 13/28, 46%) or had low voltage ECG (n = 14/28, 50%), and most patients (n = 24/28, 86%) had benign ECGs without any diagnostic ST-T changes (**Extended Data** Fig. 4). Moreover, we found no significant differences between false negatives and true positives in terms of demographics or clinical characteristics, with the exception that most false negatives had a history of a prior myocardial infarction (93% vs. 27%). The latter finding was intriguing given that our OMI model was slightly less specific in patients with known coronary artery disease (Fig. 4C).

### Screening for Any ACS Event

We further built a model to screen for any potential ACS event at first medical contact. Using the same set of ECG features, we trained and optimized a random forest classifier that denotes the likelihood of any ACS event. The model performed well during training (AUROC 0.88 [95% CI 0.87–0.90]) and generalized well during internal testing (AUROC 0.80 [95% CI 0.76–0.84]), outperforming both the commercial ECG interpretation system (AUROC 0.62 [95% CI 0.55–0.68], p< 0.001) and practicing clinicians (AUROC 0.66 [95% CI 0.59–0.72], p< 0.001) (**Extended Data** Fig. 5). On external validation, the model continued to generalize well (AUROC 0.79 [95% CI 0.76–0.81]), outperforming the commercial system (AUROC 0.68 [95% CI 0.65–0.71], p<0.001) and practicing clinicians (AUROC 0.72 [95% CI 0.69–0.74], p<0.001). Our derived risk score provided a suboptimal rule-out classification for any ACS event (sensitivity 68.2% and NPV 92.5%) but provided superior rule-in accuracy (specificity 98.9% and PPV 82.5%).

## Discussion

In this study, we developed and validated a machine learning algorithm for the ECG diagnosis of OMI in consecutive patients with chest pain recruited from multiple clinical sites in the United States. This model outperformed practicing clinicians and other commercial interpretation systems. The derived risk score provided superior rule-in and rule-out accuracy for OMI, boosting the sensitivity by 7 to 28 percentage points and the precision by 18 to 32 percentage points compared to reference standards. When combined with the judgment of experienced emergency personnel, our derived OMI risk score helped correctly reclassify one in three patients with chest pain. To our knowledge, this is the first study using machine learning methods and novel ECG features to optimize OMI detection in patients with acute chest pain and negative STEMI pattern on their baseline ECG at first medical contact.

Mapping myocardial ischemia, a problem of regional metabolic derangement, to coronary occlusion, a problem of diminished blood flow due to an atherosclerotic plaque rupture, is a complex process.^1^ Essentially, ischemia disproportionately distorts action potentials in different myocardial segments, resulting in tissue-scale currents, often called ‘injury’ currents. Prior studies have mapped significant ST-elevation to transmural injury currents associated with total coronary occlusion. This has historically driven the current paradigm dichotomy of STEMI vs. *‘others’* (any ACS other than STEMI) in determining who might benefit from emergent reperfusion therapy. However, nearly 65% of patients with ACS present with no ST-elevation on their baseline ECG,^36,37^ and among the latter group, 24%−35% have total coronary occlusion requiring emergent catheterization.^10–14^ Thus, determining who would benefit from reperfusion therapy remains an adjudicated diagnosis.

Conceptually, injury currents produced by ischemic cardiac cells are summative in nature, explaining how ST amplitude changes can get attenuated on the surface ECG (**Extended Data Fig. 6**). These injury currents, however, distort the propagation of both excitation and recovery pathways, altering the configuration of the QRS complex and the STT waveform altogether.^24^ Thus, a more comprehensive approach for the ECG detection of ischemia should focus on (1) evaluating temporal characteristics over entire waveform segments rather than the voltage at a given time point (e.g., J + 80), and (2) evaluating lead-to-lead spatial characteristics in waveform morphology rather than absolute changes in isolated ECG leads.^1^

This study has identified several ECG patterns indicative of acute coronary occlusion beyond the criteria recommended by clinical guidelines.^6^ Intriguingly, these ECG patterns overlap with those described in the literature. A consensus report in 2012 identified few ECG patterns that should be treated as STEMI equivalent during acute pain episodes: ST-depression in V1 to V3; small inverted T waves in V1 to V3; deep negative T waves in precordial leads; widespread ST-depression, and prominent positive T waves.^21^ Similar ECG patterns were also described more recently: ST-depression in V1 to V4 (versus V5-V6); reciprocal ST-depression with maximal ST-depression vector towards the apex (leads II and V5, with reciprocal STE in aVR); subtle ST-elevation; acute pathologic Q waves; hyperacute T waves; and loss of terminal S wave.^22^ Many of these expert-driven patterns rely on assessing the proportion of repolarization amplitudes or area under the QRS amplitude. They also rely heavily on the visual assessment of waveform morphology and can introduce a high degree of subjectivity and variability among ECG interpreters. We demonstrated that the machine learning models described herein not only outperform practicing clinicians in identifying OMI, but also provided an objective, observer-independent approach to quantify subtle ECG patterns associated with OMI.

Many of the data-driven features identified by our machine learning model are subtle and cannot be easily appreciated by clinical experts. T feature indices were among these most important features, including T_peak_-T_end_ interval prolongation, T wave flattening, and T wave characteristics at the inflection point preceding T_peak_ (Fig. 3A). Mechanistically, ischemic injury currents interfere with signal propagation leading to longer activation time.^38–40^ These late activation potentials lead to a loss of terminal S wave and longer recovery time, both manifesting as T wave flattening, shifted T peak, and loss of concavity at the initial T wave (Fig. 3B). These STEMI-equivalent patterns were previously described in the literature as small or negative T waves with widespread ST-depression or subtle ST-elevation.^21,22^ Another important subtle feature identified by our model was increased ventricular repolarization dispersion, measured using the ratio between the principal components of the STT waveforms (i.e., PCA metrics), the direction of the T axis, and the angle between activation and recovery pathways (e.g., total-cosine-R-to-T). Injury currents disproportionately affect the duration and velocity of repolarization across different myocardial segments,^41^ resulting in lead-to-lead variability in the morphology of the STT waveform.^23–26, 42^ These high-risk ECG patterns were previously described as a mixture of deep negative T waves and prominent / hyperacute T waves or reciprocal T wave changes.^21,22^ Despite their subtle nature, our machine learning model provided a more comprehensive, quantitative approach to evaluating this inter-lead variability in repolarization morphology.

Machine learning is well-suited to address many challenges in 12-lead ECG interpretation. Myocardial ischemia distorts the duration and amplitude of the Q wave, R peak, R’, QRS complex, ST segment, and T wave, as well as the morphology and configuration of these waveforms (e.g., upsloping, down-sloping, concavity, symmetry, notching, etc.). These distortions are lead-specific yet come with dynamic inter-lead correlations. Thus, ECG interpretation involves many complex aspects and parameters, making it a highly dimensional, decision space problem.^1^ Few experienced clinicians excel in such pattern recognition,^22^ which explains why so many OMI cases are not reperfused in a timely way; this is also why simple, rule-based commercial systems that use simple regression models are suboptimal for OMI detection. Machine learning algorithms can provide powerful tools to solve such highly dimensional, non-linear mathematical representations found in 12-lead ECG data.

Although the literature on machine learning for the ECG diagnosis of coronary disease is ubiquitous, it comes with many serious limitations. First, many studies focused on detecting the known STEMI group or other subtle ACS phenotypes^37,43–45^ rather than the critical group without ST-elevation, which is not classified as STEMI and is therefore excluded from STEMI databases. Second, most prior work used open-source ECG datasets like PTB and PTB-XL,^46^ which are highly selected datasets that focus on ECG-adjudicated diagnoses. Our unique cohorts included unselected, consecutive patients with clinical profiles and disease prevalence like that seen in real-world settings. Third, many studies used a full range of input features based on both ECG data and clinical data elements (e.g., patient history, physical exam abnormalities, laboratory values, diagnostic tests),^47–50^ which limits the applicability to real-world settings. Fourth, to our knowledge, most studies used a single derivation cohort for training and testing,^51^ without the use of an independent validation cohort. Finally, prior studies paid little attention to model explainability,^52^ shedding little light on novel markers and pathways of ischemia than what is already known. Without explanation aids of clinical meaningfulness, machine learning models for ECG interpretation would have limited clinical utility.^53^

This study has important clinical implications. Our machine learning model can help emergency personnel identify 85% more patients with critical coronary occlusion despite the absence of a STEMI pattern on the presenting ECG and without any loss in precision. Our models can also help inform care in more than 50% of patients in whom the initial assessment is indeterminate, placing 45% more patients in the low-risk group for OMI without any loss in NPV. This incremental gain in rule-in and rule-out accuracy can help re-allocate critical emergency resources to those in utmost need while optimizing the clinical workflow. This can impact numerous decisions at first medical contact, including targeted prehospital interventions, catheterization lab activation, administration of anti-ischemic therapies, hospital destination decisions, the need for medical consults, referrals for expedited diagnostic testing (e.g., echocardiogram, imaging scans), and early discharge decisions. Furthermore, until now, clinicians never had sensitive nor highly specific tools that would allow the ultra-early identification of OMI in the absence of a STEMI pattern. Such enhanced diagnostics can allow the design and implementation of prospective interventional trials to assess the therapeutic effectiveness of targeted interventions in this vulnerable group (e.g., early upstream P2Y_12_ inhibitor administration,^54^ emergent vs. delayed reperfusion therapy,^55^ glucose-insulin-potassium infusion,^56^ etc.).

Several limitations merit consideration. First, the engineered features we used for building our models are based on a manufacturer-specific software. There are known discrepancies between manufacturers in ECG preprocessing and metrics computation, which means that our models would need retraining and validation when using different software for ECG signal processing. Second, we found slight differences between the derivation and validation cohorts, specifically in terms of disease prevalence and practicing clinicians’ accuracy in ECG interpretation. These cohorts came from two different regions in the U.S., and EMS systems follow state-specific protocols. It is possible that discrepancies in EMS protocols and in-hospital practices resulted in slight differences in the types and proportions of patients that receive prehospital 12-lead ECGs, as well as in their outcome adjudications. Yet, it is reassuring that our models continued to generalize well between the study sites. Third, it is worth noting that our model for screening for “any ACS event” only boosted the performance of the rule-in arm of the derived risk score. This means that a low-risk determination by our model suggests that a given patient would unlikely have OMI, but they might still have a less subtle phenotype of NSTE-ACS that does not require reperfusion therapy. It is likely that serial ECG testing might improve the detection of this group,^43^ but this remains to be confirmed. Finally, although this study used prospective patients, all analyses were completed asynchronously with patient care. Prospective validation where OMI probabilities and decision support is provided in real time is warranted.

In conclusion, we developed and externally validated machine learning models for the ECG diagnosis of OMI in 7,313 patients with chest pain from multiple sites in the United States. The results demonstrated the superiority of machine learning in detecting subtle ischemic ECG changes indicative of OMI in an observer-independent approach. These models outperformed practicing clinicians and commercial ECG interpretation software, significantly boosting both precision and recall. Our derived OMI risk score provided superior rule-in and rule-out accuracy when compared to HEAR score, and when combined with the clinical judgment of trained emergency personnel, this score helped correctly reclassify one in three patients with chest pain. The ECG features driving our models were evaluated, providing plausible mechanistic links to myocardial injury. Future work should focus on the prospective validation where OMI probabilities and decision support is provided in real time.

## Online Methods

### Ethics Statement

The derivation cohort included prehospital data from the City of Pittsburgh Bureau of Emergency Medical Services (EMS) and in-hospital data from three tertiary care hospitals from the University of Pittsburgh Medical Center (UPMC) healthcare system: UPMC Presbyterian Hospital, UPMC Shadyside Hospital, and UPMC Mercy Hospital (Pittsburgh, Pennsylvania, USA). All consecutive eligible patients were recruited under a waiver of informed consent. This observational trial was approved by the institutional review board of the University of Pittsburgh and was registered in www.ClinicalTrials.gov (identifier #NCT04237688). The analyses described in this paper were prespecified by the trial protocol that was funded by the National Institute of Health. The first external validation cohort included data from Orange County EMS (Chapel Hill, North Carolina, USA). This study actively consented eligible patients and was approved by the institutional review board of the University of North Carolina at Chapel Hill. The second external validation cohort included data from Mecklenburg County EMS and Atrium Health (Charlotte, North Carolina, USA). Data were collected through a healthcare registry and all consecutive eligible patients were enrolled under a waiver of informed consent. This study was also approved by the institutional review board of the University of North Carolina at Chapel Hill. These two external cohorts were very comparable and were, therefore, combined into one cohort.

### Study Design & Data Collection

This was a prospective, observational cohort study. The methods for each study cohort were described in detail elsewhere.^57,58^ All study cohorts enrolled adult patients with an emergency call for non-traumatic chest pain or anginal equivalent symptoms (arm, shoulder, jaw pain, shortness of breath, diaphoresis, syncope). Eligible patients were transported by an ambulance and had at least one recorded prehospital 12-lead ECG. There were no selective exclusion criteria based on sex, race, comorbidities, or acuity of illness. For this prespecified analysis, we only included non-duplicate ECGs from unique patient encounters, and we removed patients with prehospital ECGs showing ventricular tachycardia or ventricular fibrillation (i.e., these patients are managed by ACLS algorithms). We also removed patients with confirmed prehospital STEMI, which included machine-generated ***ACUTE MI*** warning, EMS-documentation of STEMI, and medical consult for potential CATH lab activation.

Independent reviewers extracted data elements from hospital systems on all patients meeting eligibility criteria. If a prehospital ECG had no patient identifiers, we used a probabilistic matching approach to link each encounter with the correct hospital record. This previously validated data linkage protocol was based on the ECG-stamped birth date, sex, and date/time logs, as well as based on EMS dispatch logs and receiving hospital records. All probabilistic matches were manually reviewed by research specialists for accuracy. The match success rate ranged from 98.6% to 99.8%.

### Clinical Outcomes

Adjudications were made by independent reviewers at each local site after reviewing all available medical records within 30 days of the indexed encounter. Reviewers were blinded from all ECG analyses and models’ predictions. OMI was defined as coronary angiographic evidence of an acute culprit lesion in at least one of the three main coronary arteries (LAD, LCX, RCA) or their primary branches with TIMI flow grade of 0–1. TIMI flow grade of 2 with significant coronary narrowing > 70% and peak troponin of 5–10.0 ng/mL was also considered indicative of OMI.^18,22^ These adjudications were made by two independent reviewers. The Kappa coefficient statistic between the two reviewers was 0.771 (i.e., substantial agreement). All disagreements were resolved by a third reviewer.

ACS was defined per the fourth universal definition of myocardial infarction as the presence of symptoms of ischemia (i.e. diffuse discomfort in the chest, upper extremity, jaw, or epigastric area for more than 20 minutes) and at least one of the following criteria: (1) subsequent development of labile, ischemic ECG changes (e.g., ST changes, T inversion) during hospitalization; (2) elevation of cardiac troponin (i.e., > 99th percentile) during the hospital stay with rise and/or drop on serial testing; (3) coronary angiography demonstrating greater than 70% stenosis, with or without treatment; and/or (4) functional cardiac evaluation (stress testing) that demonstrates ECG, echocardiographic, or radionuclide evidence of focal cardiac ischemia.^6^ Patients with type 2 MI and pre-existing subacute coronary occlusion were labeled as negative for ACS and OMI. This included around 10% of patients with positive troponin but with no rise and/or drop in concentration on serial testing (i.e., chronic leak) or with troponin leak attributed to noncoronary occlusive conditions such as pericarditis. On a randomly selected small subset of patients (n=1,209), the Kappa coefficient statistic for ACS adjudication ranged from 0.846 to 0.916 (i.e., substantial to perfect agreement).

### ECG Methods

Prehospital ECGs were obtained in the field by paramedics as part of routine care. ECGs were acquired using either Heart Start MRX (Philips Healthcare) or LIFEPAK-15 (Physio-Control Inc.) monitor-defibrillator devices. All digital 12-lead ECGs were acquired at a sampling rate of 500 s/s (0.05–150 Hz) and transmitted to the respective EMS agency and receiving hospital. Digital ECG files were exported in XML format and stored in a secondary server at each local site. ECG images were de-identified and manually annotated by independent reviewers or research specialists; ECGs with poor quality or missing leads were removed from the study. Next, digital XML files were transmitted to the Philips Advanced Algorithm Research Center for offline analysis (Cambridge, Massachusetts, USA).

ECG featurization was described in detail elsewhere.^19^ Briefly, ECG signal preprocessing and feature extraction were performed using a manufacturer-specific software (Philips DXL diagnostic 12/16 lead ECG analysis program). ECG signals were first preprocessed to remove noise, artifacts, and baseline wander. Ectopic beats were removed, and median beats were calculated for each lead. Next, we used the root mean square (RMS) signal to identify global waveform fiducials, including the onset, offset, and peak of the P wave, QRS complex, and T wave. Lead-specific fiducials were then identified to further segment individual waveforms into Q, R, R, *S, S*, and J point.

We then computed a total of 554 ECG features based on (1) the amplitude, duration, area, slope and/or concavity of global and lead-specific waveforms; (2) the QRS and T axes and angles in the frontal, horizontal, spatial, XY, XZ, and YZ planes, including directions at peak, inflection point, and initial / terminal loops; (3) eigenvalues of the principal components of orthogonal ECG leads (I, II, V1-V6), including PCA ratios for individual ECG waveform segments; and (4) T loop morphology descriptors. Features with zero distribution were removed to prevent representation bias.

Next, we identified an optimal parsimonious list of the most important ECG features that are mechanistically linked to cardiac ischemia as described in detail elsewhere.^19^ Briefly, to prevent omitted-feature bias, we used a hybrid approach that combines domain knowledge with a data-driven strategy. Clinical scientists initially reviewed a list of 554 features and marked the ones that are known to correlate with cardiac ischemia. This list was then expanded by supplemental features identified by data-driven algorithms (e.g., recursive feature elimination and LASSO). The clinical scientists then reviewed the expanded list to examine feature pairs with high collinearity and retained the subset of features that are complementary and can serve as plausible markers of ischemia. This approach eventually yielded a subset of 73 features that was shown to boost classification performance.^19^

### Machine Learning Methods

We followed best practices recommended by “ROBUST-ML” and “ECG-AI stress test” checklists to design and benchmark our machine learning algorithms.^52,59^ To prevent measurement bias, ECG features were manually reviewed to identify erroneous calculations. Physiologically plausible outliers were replaced with ±3 SD. On average, each feature had a 0.34% missingness rate (range 0.1 % to 1.6%). Thus, we imputed missing values with the mean, median, or mode of that feature after consultation with clinical experts. ECG metrics were then z-score normalized and used as input features in machine learning models. The derivation and validation datasets were cleaned independently to prevent data leakage. Both cohorts were recruited over the same time window, suggesting the lack of temporal bias. To prevent potential mismatch with intended use, input features for model development included only ECG data plus the machine-stamped age. No other clinical data were used for model building.

We randomly split the derivation cohort into an 80% training set and a 20% internal testing set. On the training set, we fit 10 machine learning classifiers: regularized logistic regression, linear discriminant analysis, support vector machine, Gaussian Naive Bayes, random forest, gradient boosting machine, extreme gradient boosting, stochastic gradient descent logistic regression, k-nearest neighbors, and artificial neural networks. Each classifier was optimized over 10-fold cross validation to finetune hyperparameters. After selecting optimal hyperparameters, models were re-trained on the entire training subset to derive final weights and create a lockout model to evaluate on the holdout test set. We calibrated our classifiers to produce a probabilistic output which can be interpreted as a confidence level (probability risk score). Trained models were compared using the AUROC curve with Wilcoxon signed-rank test for pairwise comparisons. ROC-optimized cutoffs were chosen using Youden index, and classifications on confusion matrix were compared using McNemar’s test.

The random forest classifier (RF) achieved high accuracy on the training set (low bias) with a relatively small drop in performance on the test set (low variance), indicating an acceptable bias-variance tradeoff and low risk of overfitting (**Extended Data Fig. 7**). Although the support vector machine (SVM) model had lower variance on the test set, when compared with the RF model, there were no significant differences in AUROC (Delong’s test) or their binary classifications (McNemar’s test). Moreover, there were no differences between the RF and SVM models in terms of Kolmogorov-Smirnov goodness-of-fit (0.716 vs. 0.715) or the Gini purity index (0.82 vs. 0.85). Due to its scalability and intuitive architecture, we chose the probability output of the RF model to build our derived OMI score. We generated density plots of these probability scores for positive and negative classes and selected classification thresholds for low, intermediate, and high-risk groups based on prespecified NPV > 0.99 and TPR > 0.50. Finally, we used the lock-out random forest classifier to generate probability scores and risk classes on the completely unseen external validation cohort. The code to generate probability scores is included with the supplemental materials of this manuscript.

### Reference Standard

To reduce the risk of evaluation bias, we benchmarked our machine learning models against multiple reference standards. First, we used a commercial, FDA-approved, ECG interpretation software (Philips DXL diagnostic algorithm) to denote the likelihood of ischemic myocardial injury. This likelihood (yes/no) was based on a composite of the followings: (1) diagnostic codes for “>>>Acute MI<<<“, including descriptive statements that denote “acute”, “recent”, “age indeterminate”, “possible” or “probable”; and (2) diagnostic codes for “>>>Acute Ischemia<<<“, including descriptive statements that denote “possible”, “probable”, or “consider”. Diagnostic statements that denoted “old” [infarct], “nonspecific” [ST depression], or “secondary to” [LVH or high heart rate] were excluded from this composite reference standard.

We also used practicing clinicians’ overread of ECGs to denote the likelihood of ischemic myocardial injury (yes/no). Independent physician reviewers annotated each 12-lead ECG image as per the fourth universal definition of MI criteria,^6^ including two contiguous leads with ST-elevation (> 0.2 mV for V2-V3 in men > 40 years and > 2.5 mm in men < 40 years; > 0.15 mV for V2-V3 in women; or > 0.1 mV in other leads) or ST-depression (new horizontal or down-sloping depression > 0.05 mV); with or without T wave inversion (> 0.1 mV in leads with prominent R wave or R/S ratio > 1). Reviewers were also prompted to use their clinical judgment to identify highly suspicious ischemic changes (e.g., reciprocal changes, hyperacute T waves), as well as to account for potential confounders (e.g., bundle branch blocks, early repolarization). On a randomly selected subset of patients in the derivation cohort (n=1,646), the Kappa coefficient statistic between two emergency physicians who interpreted the ECGs was 0.568 (i.e., moderate agreement). Similarly, on a randomly selected subset of patients in the external validation cohort (n=375), the Kappa coefficient statistic between the two board-certified cardiologists who interpreted the ECGs was 0.690 (i.e., substantial agreement).

Finally, we compared our derived risk score against the HEART risk score. This score is commonly used in US hospitals and it has been well-validated for triaging patients in the emergency department.^60^ The HEART score is based on the patient’s **H**istory at presentation, **E**CG interpretation, **A**ge, **R**isk factors, and initial **T**roponin values (range 0–10). This score places patients in low (0–3), intermediate (4–6), and high-risk (7–10) groups. Given that troponin results are not usually available at first medical contact, we used a modified HEAR score after dropping the **T**roponin values, which has also been previously validated for use by paramedics prior to hospital arrival.

### Statistical Analysis

Descriptive statistics were reported as mean ± standard deviation or n (%). Missing data was assessed for randomness and was handled during ECG feature selection (see Machine Learning Methods section above). Normality of distribution was assessed prior to hypothesis testing where deemed necessary. ECG features were z-score normalized as part of standard input architectures for machine learning models. Comparisons between cohorts were performed using chi-square (for discrete variables) and independent samples t-test or Mann-Whitney U test (for continuous variables). The level of significance was set at alpha 0.05 for two-tailed hypothesis testing where applicable.

All diagnostic accuracy values were reported as per STARD recommendations (Reporting Guidelines for Diagnostic Accuracy Studies). We reported classification performance using AUROC curve, sensitivity (recall), specificity, PPV (precision), and NPV, along with 95% confidence interval (CI) where applicable. For 10-fold cross validation, we compared the multiple classifiers using the Wilcoxon signed-rank test (for AUROC curves) and McNemar’s test (for confusion matrices). We derived low-, intermediate-, and high-risk categories for the final classifier using Kernel density plot estimates between classes. The adequacy of these risk classes was evaluated using Log-rank chi-square of accumulative risk for clinically important outcomes over the length of stay during the indexed admission.

For assessing the incremental gain in classification performance, we compared the AUROC of the final model against reference standards using DeLong’s test. For ease of comparison, the confidence bounds for AUROC of the reference standards (commercial system and practicing clinicians) were generated using 1000 bootstrap samples. We then computed the Net Reclassification Improvement (NRI) index of our model against the HEAR score during the initial assessment at first medical contact. We used logistic regression to identify the independent predictive value of OMI risk classes. We used variables significant in univariate analysis and then built multivariate models with stepwise backward selection method using Wald chi-square criteria. We reported odds ratios with 95% CI for all significant predictors. All analyses were completed using Python v3.8.5 and SPSS v24.

## Figures and Tables

**Table 1:** Baseline demographic and clinical characteristics

	Derivation & Testing Cohort (n = 4,026)	external validation cohort (n = 3,287)
age (years)	59 ± 16 (18 – 102)	60 ±15 (21 −100)
sex	2,122 (53%)	1,814 (55%)
Male	1,904 (47%)	1,473 (45%)
Female		
race	1,698 (42%)	1,326 (40%)
White	1,328 (33%)	1,544 (47%)
Black	52 (1.3%)	40 (1 %)
Others	948 (24%)	377 (12%)
Unknown		
ethnicity	3,043 (76%)	2,850 (87%)
Not Hispanic	19 (1%)	116 (3.5%)
Hispanic / Latino	964 (23%)	321 (9.5%)
Unknown		
Past medical history	2,767 (69%)	2,090 (64%)
Hypertension	1,146 (29%)	1,067 (33%)
Diabetes	1,520 (38%)	1,376 (42%)
High cholesterol	1,244 (31%)	802 (25%)
Current smoker	1,388 (35%)	964 (30%)
Known CAD	930 (23%)	929 (29%)
Prior MI	963 (24%)	134 (4%)
Prior PCI	357 (10%)	470 (14%)
Prior CABG		
ECG & Lab findings	3,496 (87%)	2,614 (80%)
Sinus rhythm	354 (9%)	352 (11%)
Atrial fibrillation	94 (2.3%)	114 (3.5%)
Left BBB	237 (5.9%)	215 (6.6%)
Right BBB	383 (9.5%)	467 (14.2%)
ECG-LVH	330 (8.2%)	736 (22.4%)
cTnl positive (initial)	729 (18.1%)	1,177 (35.8%)
cTnl positive (serial testing)		
Medical therapy	300 (7.5%)	245 (7.5%)
pci (ANY STENT)	144 (3.6%)	157 (4.8%)
*Emergent PCI (< 90 MIN)*	91 (2.3%)	94 (2.9%)
*Total LAD occlusion*	63 (1.6%)	88 (2.7%)
*Total LCX occlusion*	101 (2.5%)	102 (3.1%)
*Total RCA occlusion*	34 (0.8%)	30 (0.9%)
CABG		
study outcomes	550 (13.7%)	537 (16.3%)
confirmed acs	210 (5.2%)	209 (6.4%)
*OMI*	240 (6.0%)	220 (6.7%)
*Other Acute MI (NOM)*	100 (2.5%)	108 (3.3%)
*Unstable Angina*	137 (3.4%)	111 (3.4%)
30-day cv death		

Values are mean ± SD (min-max) or n (%); CAD: coronary artery disease; MI: myocardial infarction; BBB: bundle branch block; LVH: left ventricular hypertrophy; PCI: percutaneous coronary intervention; LAD: left anterior descending artery; LCX: left circumflex artery; RCA: right coronary artery; CABG: coronary artery bypass graft; OMI: occlusion MI; NOMI: non-occlusion MI; CV: cardiovascular.

## Data Availability

Input features and output outcomes used for deriving and testing the machine learning models are provided in CSV format along with this article.
